# Cammidge's Pancreatic Reaction

**Published:** 1908-06-06

**Authors:** 


### Pathology.

### CAMMIDGE'S PANCREATIC REACTION.

In our issue of May 23 last we gave a full account of Cammidge's pancreatic reaction in its original form, together with conclusions as to its value in that form, based upon results found by Mr. Cassidy and published in a recent issue of the St. Thomas's Hospital Reports. It is possible that there are workers who still use the test in its 1904 shape; there have, however, been certain modifications and improvements in the test since that time, and we are indebted to Dr. Cammidge himself for a letter to that effect. We regret that, by the omission of reference to his later work, an injustice should have been done to the author's test. Original articles upon the subject will be found in the "Transactions of the Royal Medical and Chirurgical Society," Vol. 89; in the "British Medical Journal" for March 10, 1906, May 19, 1906, and April 11, 1908; in the "Edinburgh Medical Journal" for February 1907; in "Surgery, Gynæcology, and Obstetrics" for September 1907 and January 1908; in Keen's "System of Surgery"; in the "Clinical Journal" for April 1, 1908; and in "The Pancreas, its Surgery and Pathology," by Mayo Robson and Cammidge.

The general conclusion arrived at by various workers was, as we stated in our former article, that whereas the original pancreatic reaction of the urine consisted of three parts—namely, reaction A, reaction B, and the degree of solubility of the crystals obtained by A or B—only reaction A seemed to be of real clinical importance. It is a great simplification of the test to have got rid of the necessity for investigating reaction B, and the degree of solubility of the crystals in each case, and to have reduced the procedure to a consideration of one reaction only. The advances and improvement in the test have been in the direction of perfecting reaction A.

As Dr. Cammidge found and pointed out before the Royal Medical and Chirurgical Society in 1906, examination of the phenylhydrazin precipitate derived from the urine in cases of pancreatic inflammation, after treatment with hydrochloric acid (original reaction A), showed that it consisted of two parts, one a phenylhydrazin compound of glycuronic acid and the other the osazone of a sugar. Although there is reason to believe that the excretion of glycuronic acid is increased in pancreatitis, an augmentation of the output occurs in so many other pathological conditions that no helpful diagnostic method could be based upon this in the present state of our knowledge. Further investigation of the precipitate obtained from the urine after treatment with mercuric chloride (original reaction B) showed that it consisted entirely, or almost entirely, of a glycuronic acid compound of phenylhydrazin, so that the difference noticed between A and B reactions in characteristic cases of pancreatic disease was probably dependent on some substance other than glycuronic acid. If, therefore, the glycuronic acid could be removed previous to employing the phenylhydrazin test, the result might be expected to be more definite, and the necessity for two comparative tests be done away with. This was found to be possible by taking advantage of the fact that glycuronic acid is precipitated by basic lead acetate from acid solutions, while the sugars are said to be thrown out only when the reaction of the fluid is alkaline. Many minor difficulties had to be overcome before a practical working method giving reliable results could be arrived at, and a large number of urines had to be examined by the old and new tests before their comparative value could be ascertained. The result was that the newer method was found to be a distinct improvement as regards the definiteness of the data upon which an opinion could be formed. In Dr. Cammidge's own words, the newer form of the reaction is as follows:—

A specimen of the twenty-four hours' urine, or of the mixed morning and evening secretions, is filtered several times through the same filter-paper and examined for albumen, sugar, bile, urobilin, and indican. A quantitative estimation of the chlorides, phosphates, and urea is also made, and the centrifugalised deposit from the urine examined microscopically for calcium oxalate crystals. If the urine is found to be free from sugar and albumen, and is acid in reaction, 1 c.c. of strong hydrochloric acid (sp. gr. 1.16) is mixed with 20 c.c. of the clear filtrate, and the mixture gently boiled on the sand-bath in a small flask, having a long-stemmed funnel in the neck to act as a condenser. After ten minutes' boiling the flask is well cooled in a stream of water, and the contents made up to 20 c.c. with cold distilled water. The excess of acid present is neutralised by slowly adding 4 grammes of lead carbonate. After standing for a few minutes to allow of the completion of the reaction, the flask is again cooled in running water, and the contents filtered through a well-moistened close-grained filter-paper until a perfectly clear filtrate is secured. The filtrate is then well shaken with 4 grammes of powdered tribasic lead acetate, and the resulting precipitate removed by filtration, an absolutely clear filtrate being obtained by repeating the filtration several times if necessary. Since the large amount of lead now in solution would interfere with the subsequent steps of the experiment, it is removed either by treatment with a stream of sulphuretted hydrogen or, what has been found to be equally satisfactory and less disagreeable, by precipitating the lead as sulphate. For this purpose the clear filtrate is well shaken with 2 grammes of finely-powdered sodium sulphate, the mixture heated to the boiling-point, then cooled to as low a temperature in a stream of cold water, and the white precipitate removed by careful filtration; 10 c.c. of the perfectly clear transparent filtrate are made up to 18 c.c. with distilled water, and added to 0.8 gramme of phenylhydrazin hydrochlorate, 2 grammes of powdered sodium acetate, and 1 c.c. of 50 per cent. acetic acid contained in a small flask fitted with a funnel condenser. The mixture is boiled on a sand-bath for ten minutes, and then filtered hot through a filter-paper moistened with hot water into a test-tube provided with a 15 c.c. mark. Should the filtrate fail to reach the mark, it is made up to 15 c.c. with hot distilled water, but after a little practice this is rarely found to be necessary, for it is possible to regulate the boiling process so that the final result always comes out at between 15 and 16 c.c. In well-marked cases of pancreatic inflammation a light yellow flocculent precipitate should form in a few hours, but it may be necessary to leave the preparation to stand overnight before a deposit occurs. Under the microscope the precipitate is seen to consist of long, light yellow, flexible, hair-like crystals, arranged in sheaves which, when irrigated with 33 per cent. sulphuric acid, melt away and disappear in ten to fifteen seconds after the acid first touches them. The precipitate should always be examined microscopically, as it may be difficult to determine the characters of a small deposit with the naked eye, and so cases giving only a slight reaction may be overlooked. To exclude traces of sugar, undetected by the preliminary reduction tests, a control experiment is carried out by treating 20 c.c. of the urine in the same way as in the test described, excepting for the addition of the hydrochloric acid.

It is quite clear that, although Cammidge's pancreatic reaction has been reduced by the above procedure from three separate processes to a single one, it is of too great a complexity for general use in practitioners' own hands. Urines to be investigated for the reaction need to be sent to some clinical laboratory where all the facilities for carrying it out are to hand. It should be mentioned that, even when the procedure recommended is faithfully followed, there may at first be some difficulty in obtaining positive reactions. Dr. Chalmers Watson, for example, writes that "at the outset of my observations I experienced some difficulty with the test, and failed to get any reaction. This failure may have been due to faulty chemicals employed. At a later date Dr. Cammidge kindly demonstrated to me the end reaction, and subsequently I have experienced no special difficulty with the test. The present series of analyses was carried out by a trained assistant in the laboratory of the Royal College of Physicians (Edinburgh), the end reaction being determined by myself, a microscopic examination being made in every instance."

Many results of the test in practice have been and are being published. It will probably be some little time before the precise limitations of the test have been determined; it is even possible that the test itself may be modified and perhaps simplified as time goes on. Meanwhile it seems to be increasingly clear that when, in a given case, the reaction is negative the pancreas is not at all likely to be the cause of the symptoms; whereas when the reaction is positive some lesion of the pancreas is very probably present.

Dr. Cammidge does not contend that at all times and under all conditions the results given by the improved method of examining the urine are indicative of the presence or absence of pancreatic inflammation; but the evidence available suggests that it may be of considerable assistance in diagnosis, so that the 50 per cent. of cases of pancreatic disease now said to be only recognised post mortem might be reduced to a more creditable figure.

Dr. Chalmers Watson (*loc. cit.* 1908) states as his general conclusions from independent observations that "My results confirm the conclusion arrived at by Mayo Robson and Cammidge that there is a definite and important relationship between the pancreatic reaction in the urine and disease of the pancreas. My results differ from those of Cammidge in so far that I have recorded a higher percentage of positive results in cases similar to those that may have been included in that author's list of control cases. Cammidge found in a group of cases described as miscellaneous—other than those having a direct surgical interest in relation to disease of bile-duct and pancreas—that four only out of 92 gave a positive reaction. The higher percentage of positive results recorded in my series may possibly be due to the fact that I made use of many serious medical cases of a nature commonly associated with advanced arterial disease, and chronic catarrhal conditions in the intestinal tract, conditions that are frequently attended with degenerative or inflammatory changes in the pancreas and other glands. My results lead me to divide the cases in which the pancreatic reaction was present in my series into the following groups:—

1. A group in which there was definite clinical or pathological evidence of serious organic disease of the pancreas; for example, acute and chronic pancreatitis, usually associated with disease of the bile ducts.

2. A group in which the reaction in the urine is associated with pronounced arterial sclerosis, a condition usually accompanied by more or less sclerosis in different glands.

3. A group in which the reaction is dependent on congestion and catarrhal conditions of the gland ducts and substance, with associated toxæmia—for example, advanced heart disease, appendicitis, pneumonia, malaria, and the like.

This division is, it is hardly necessary to add, somewhat arbitrary, as most cases are in all probability of mixed origin. If these facts are kept in view, and this urinary reaction is carefully studied along with the other clinical features of a case, I have no hesitation in saying that this new test will prove of great value both to physicians and surgeons in the diagnosis and treatment of pancreatic disease."

Dr. Cammidge points out the importance of examining the fæces for undigested muscle fibre and for fat in cases in which it is uncertain whether the pancreatic affection is inflammatory or malignant. In one such case, in which the urinary pancreatic reaction was positive, the excretion of chlorides was much diminished, while the urea and phosphates were approximately normal. The fæces were found to be acid in reaction, to contain a large amount of undigested muscle-fibre, and to yield not a trace of stercobilin. An estimation of the fats showed a total of 64.7 per cent., of which 56.3 per cent. was "neutral fat" and only 8.4 "fatty acid." The laboratory results, therefore, taken as a whole, confirmed the clinical diagnosis and pointed to carcinoma of the pancreas with secondary inflammation.

It is necessary to emphasise the fact that the laboratory investigations require a considerable amount of care and practice, and that it is necessary to weigh all the evidence obtainable in forming a conclusion.

